# Supplementary material for: Modeling glioblastoma heterogeneity as a dynamic network of cell states
Source: Mol Syst Biol. 2021 Sep 16;17(9):e10105. doi: 10.15252/msb.202010105 (PMC8444284; doi:10.15252/msb.202010105)
Supplement: Supplementary file 6 — Source Data for Figure 5 [file MSB-17-e10105-s004.zip › Figure5A_sourcedata/GSEA_3071/nosigdb_stateI.GseaPreranked.1622795145664/LARSSON_STATE_4.html]

Details for gene set LARSSON\_STATE\_4[GSEA]

|  || Dataset | state13071 |
| Phenotype | NoPhenotypeAvailable |
| Upregulated in class | na\_neg |
| GeneSet | LARSSON\_STATE\_4 |
| Enrichment Score (ES) | -0.73204666 |
| Normalized Enrichment Score (NES) | -3.7963247 |
| Nominal p-value | 0.0 |
| FDR q-value | 0.0 |
| FWER p-Value | 0.0 |
Table: GSEA Results Summary

  

Fig 1: Enrichment plot: LARSSON\_STATE\_4      
 Profile of the Running ES Score & Positions of GeneSet Members on the Rank Ordered List

  

| PROBE | GENE SYMBOL | GENE\_TITLE | RANK IN GENE LIST | RANK METRIC SCORE | RUNNING ES | CORE ENRICHMENT || 1 | RAN |  |  | 1907 | -0.306 | -0.5543 | No |
| 2 | KPNA2 |  |  | 1944 | -0.312 | -0.5608 | No |
| 3 | NUCKS1 |  |  | 2278 | -0.368 | -0.6535 | No |
| 4 | CKS1B |  |  | 2332 | -0.380 | -0.6641 | No |
| 5 | UHRF1 |  |  | 2404 | -0.393 | -0.6799 | No |
| 6 | H2AFV |  |  | 2431 | -0.398 | -0.6823 | No |
| 7 | CKS2 |  |  | 2466 | -0.404 | -0.6871 | No |
| 8 | H2AFZ |  |  | 2572 | -0.433 | -0.7122 | No |
| 9 | ANP32B |  |  | 2593 | -0.438 | -0.7124 | No |
| 10 | DUT |  |  | 2640 | -0.450 | -0.7200 | No |
| 11 | MND1 |  |  | 2682 | -0.468 | -0.7260 | Yes |
| 12 | SNRPD1 |  |  | 2701 | -0.471 | -0.7252 | Yes |
| 13 | CENPN |  |  | 2708 | -0.474 | -0.7208 | Yes |
| 14 | RPA3 |  |  | 2730 | -0.480 | -0.7208 | Yes |
| 15 | CENPF |  |  | 2761 | -0.490 | -0.7232 | Yes |
| 16 | TUBA1B |  |  | 2785 | -0.498 | -0.7235 | Yes |
| 17 | DTYMK |  |  | 2798 | -0.501 | -0.7205 | Yes |
| 18 | HMGB1 |  |  | 2806 | -0.503 | -0.7161 | Yes |
| 19 | LSM5 |  |  | 2820 | -0.509 | -0.7133 | Yes |
| 20 | SIVA1 |  |  | 2822 | -0.510 | -0.7070 | Yes |
| 21 | ASF1B |  |  | 2824 | -0.510 | -0.7007 | Yes |
| 22 | TOP2A |  |  | 2842 | -0.517 | -0.6990 | Yes |
| 23 | NUDT1 |  |  | 2859 | -0.524 | -0.6969 | Yes |
| 24 | PTTG1 |  |  | 2895 | -0.540 | -0.7002 | Yes |
| 25 | CENPH |  |  | 2945 | -0.569 | -0.7072 | Yes |
| 26 | KIF4A |  |  | 2953 | -0.574 | -0.7018 | Yes |
| 27 | PIMREG |  |  | 2971 | -0.583 | -0.6993 | Yes |
| 28 | SMC2 |  |  | 2984 | -0.592 | -0.6951 | Yes |
| 29 | TPX2 |  |  | 3006 | -0.606 | -0.6935 | Yes |
| 30 | LSM3 |  |  | 3037 | -0.625 | -0.6942 | Yes |
| 31 | PHF19 |  |  | 3060 | -0.643 | -0.6923 | Yes |
| 32 | ALYREF |  |  | 3068 | -0.649 | -0.6860 | Yes |
| 33 | FANCI |  |  | 3074 | -0.656 | -0.6790 | Yes |
| 34 | CDC45 |  |  | 3081 | -0.661 | -0.6722 | Yes |
| 35 | GTSE1 |  |  | 3083 | -0.663 | -0.6639 | Yes |
| 36 | TMPO |  |  | 3088 | -0.666 | -0.6565 | Yes |
| 37 | MAD2L1 |  |  | 3099 | -0.675 | -0.6507 | Yes |
| 38 | LSM4 |  |  | 3115 | -0.688 | -0.6462 | Yes |
| 39 | DBF4 |  |  | 3117 | -0.690 | -0.6376 | Yes |
| 40 | LMNB1 |  |  | 3121 | -0.692 | -0.6295 | Yes |
| 41 | SMC4 |  |  | 3126 | -0.699 | -0.6216 | Yes |
| 42 | CENPK |  |  | 3127 | -0.700 | -0.6126 | Yes |
| 43 | MCM5 |  |  | 3132 | -0.706 | -0.6046 | Yes |
| 44 | GMNN |  |  | 3140 | -0.719 | -0.5974 | Yes |
| 45 | KIF15 |  |  | 3142 | -0.723 | -0.5884 | Yes |
| 46 | CDK1 |  |  | 3149 | -0.727 | -0.5807 | Yes |
| 47 | HNRNPD |  |  | 3157 | -0.738 | -0.5732 | Yes |
| 48 | MKI67 |  |  | 3158 | -0.738 | -0.5637 | Yes |
| 49 | ORC6 |  |  | 3159 | -0.738 | -0.5541 | Yes |
| 50 | TK1 |  |  | 3162 | -0.745 | -0.5451 | Yes |
| 51 | STMN1 |  |  | 3171 | -0.751 | -0.5377 | Yes |
| 52 | MCM3 |  |  | 3182 | -0.761 | -0.5308 | Yes |
| 53 | BRCA1 |  |  | 3185 | -0.765 | -0.5215 | Yes |
| 54 | SGO1 |  |  | 3202 | -0.783 | -0.5161 | Yes |
| 55 | BIRC5 |  |  | 3208 | -0.793 | -0.5073 | Yes |
| 56 | FBXO5 |  |  | 3212 | -0.797 | -0.4979 | Yes |
| 57 | RRM1 |  |  | 3215 | -0.801 | -0.4881 | Yes |
| 58 | UBE2C |  |  | 3225 | -0.812 | -0.4803 | Yes |
| 59 | NUSAP1 |  |  | 3237 | -0.825 | -0.4728 | Yes |
| 60 | TMSB15A |  |  | 3241 | -0.833 | -0.4629 | Yes |
| 61 | HMGN2 |  |  | 3242 | -0.833 | -0.4522 | Yes |
| 62 | RFC2 |  |  | 3245 | -0.839 | -0.4419 | Yes |
| 63 | CENPM |  |  | 3269 | -0.881 | -0.4373 | Yes |
| 64 | CHAF1A |  |  | 3270 | -0.882 | -0.4259 | Yes |
| 65 | H2AFX |  |  | 3282 | -0.898 | -0.4175 | Yes |
| 66 | E2F1 |  |  | 3289 | -0.911 | -0.4075 | Yes |
| 67 | RANBP1 |  |  | 3290 | -0.911 | -0.3957 | Yes |
| 68 | CDKN3 |  |  | 3293 | -0.916 | -0.3845 | Yes |
| 69 | ZWINT |  |  | 3296 | -0.925 | -0.3731 | Yes |
| 70 | PCNA |  |  | 3319 | -0.963 | -0.3671 | Yes |
| 71 | UBE2T |  |  | 3330 | -0.979 | -0.3574 | Yes |
| 72 | MYBL2 |  |  | 3340 | -1.006 | -0.3470 | Yes |
| 73 | MCM4 |  |  | 3341 | -1.006 | -0.3340 | Yes |
| 74 | EZH2 |  |  | 3342 | -1.015 | -0.3209 | Yes |
| 75 | DEK |  |  | 3344 | -1.025 | -0.3080 | Yes |
| 76 | RAD51AP1 |  |  | 3345 | -1.030 | -0.2946 | Yes |
| 77 | PBK |  |  | 3354 | -1.046 | -0.2835 | Yes |
| 78 | MCM10 |  |  | 3356 | -1.050 | -0.2702 | Yes |
| 79 | ESCO2 |  |  | 3367 | -1.088 | -0.2591 | Yes |
| 80 | NCAPG |  |  | 3370 | -1.098 | -0.2455 | Yes |
| 81 | MCM7 |  |  | 3384 | -1.147 | -0.2344 | Yes |
| 82 | GINS2 |  |  | 3386 | -1.156 | -0.2198 | Yes |
| 83 | DHFR |  |  | 3387 | -1.159 | -0.2048 | Yes |
| 84 | HMGB2 |  |  | 3390 | -1.184 | -0.1901 | Yes |
| 85 | CLSPN |  |  | 3391 | -1.184 | -0.1748 | Yes |
| 86 | UBE2S |  |  | 3396 | -1.205 | -0.1604 | Yes |
| 87 | CENPU |  |  | 3398 | -1.230 | -0.1448 | Yes |
| 88 | HIST1H1E |  |  | 3401 | -1.237 | -0.1294 | Yes |
| 89 | PCLAF |  |  | 3409 | -1.265 | -0.1151 | Yes |
| 90 | ATAD2 |  |  | 3415 | -1.278 | -0.1001 | Yes |
| 91 | RRM2 |  |  | 3420 | -1.297 | -0.0845 | Yes |
| 92 | HIST1H1C |  |  | 3427 | -1.335 | -0.0690 | Yes |
| 93 | TYMS |  |  | 3436 | -1.391 | -0.0534 | Yes |
| 94 | HIST1H1D |  |  | 3466 | -1.743 | -0.0393 | Yes |
| 95 | HIST1H1B |  |  | 3467 | -1.770 | -0.0165 | Yes |
| 96 | HIST1H4C |  |  | 3493 | -2.248 | 0.0053 | Yes |
Table: GSEA details [plain text format]

  

Fig 2: LARSSON\_STATE\_4: Random ES distribution      
 Gene set null distribution of ES for **LARSSON\_STATE\_4**
